# Meteorological Table

**Published:** 1811-07

**Authors:** 


### METEOROLOGICAL TABLE.

### *From May 27, to June 26.*

| | D | | Therm. | | | Barom. | | | | Hygrom. dry——damp | | | | Atmospherical Variations |
|---|---|---|---|---|---|---|---|---|---|---|---|---|---|---|
| | 28 | 65 | — | 57 | $29^{6}$ | — | —$^{7}$ | | | | 30 | 60 | 15 | SW ... F .. — ... — .... |
| | 29 | 58 | 57 | 55 | —$^{7}$ | — | —$^{9}$ | | | | 24 | 3 | 27 | W ... C ... R ... F .... |
| ◐ | 30 | 55 | 72 | 61 | —$^{9}$ | — | —$^{7}$ | | | | 30 | 9 | 10 | W .. F ... — .... — .... |
| | 31 | 59 | 65 | 60 | —$^{5}$ | — | — | | | | 30 | — | 32 | N . R ... F .. R .. F ... |
| | 1 | 58 | 64 | 60 | —$^{6}$ | — | —$^{7}$ | | | | 33 | 19 | 25 | S .. C . F ... R . F ... |
| | 2 | 59 | 60 | 56 | —$^{5}$ | — | — | | | | 33 | 45 | 42 | V .. R ... — ... F ... R .. |
| | 3 | 56 | 65 | 55 | —$^{7}$ | — | —$^{9}$ | | | | 44 | 55 | 25 | W ... NW. C ...F.... F .... |
| | 4 | 57 | 65 | 50 | —$^{9}$ | — | —$^{8}$ | | | | 30 | 20 | 26 | W.. NW .F ... — .... —... |
| | 5 | 58 | 57 | 55 | —$^{6}$ | — | —$^{7}$ | | | | 57 | 52 | 49 | SW... R ... F .. R ... F... |
| ○ | 6 | 58 | 67 | 57 | —$^{8}$ | — | — | | | | 48 | 10 | 26 | SW ... F .. — .... — .... |
| | 7 | 59 | 68 | 61 | —$^{9}$ | — | 30 | | | | 45 | 33 | 40 | SW .. F .. C .. F .... |
| | 8 | 64 | 76 | 56 | $29^{9}$ | — | 30 | | | | 40 | 30 | 20 | SE ... F ... R .. F ... R ... |
| | 9 | 59 | 70 | 57 | —$^{1}$ | — | —$^{2}$ | | | | 40 | 35 | 55 | W .. F .... — .... — .... |
| | 10 | 59 | 72 | 60 | —$^{2}$ | — | —$^{1}$ | — | — | 10 | 12 | 2 | — | E .. F .... — .... — .... |
| | 11 | 60 | 65 | 57 | $29^{9}$ | — | — | — | 8 | — | 10 | — | 5 | SW .. F ... C .. F .... |
| | 12 | 59 | 65 | 54 | —$^{3}$ | — | 30 | | | | 17 | 40 | 46 | W .. F .... R . F .... — .... |
| ◑ | 13 | 53 | 61 | 55 | —$^{1}$ | — | — | — | 7 | — | 7 | — | 3 | W .. F ... — .... — .... |
| | 14 | 56 | 67 | 59 | 30 | — | — | — | 24 | 12 | 10 | — | — | SW .. F .... — ... — .... |
| | 15 | 60 | 72 | 61 | $29^{8}$ | — | 30 | 4 | 38 | 10 | | | | W .. F ... — .... — .... |
| | 16 | 60 | 70 | 61 | $29^{9}$ | — | 30 | — | 16 | 7 | 1 | — | — | W . C .. F .. R . F .... |
| | 17 | 58 | 72 | 62 | —$^{1}$ | — | —$^{3}$ | 3 | 20 | 3 | | | | N . F ... — .... — .... |
| | 18 | 60 | 71 | 63 | —$^{3}$ | — | —$^{2}$ | — | 8 | 12 | 6 | — | — | E .. C ... F .... — .... |
| | 19 | 60 | 76 | 66 | —$^{2}$ | — | 30 | 3 | 30 | 1[illegible] | | | | W . F ... — .... C .... |
| ● | 20 | 61 | 60 | 56 | $29^{7}$ | — | —$^{6}$ | 13 | 15 | 10 | | | | N .. W .. C ... — .... — .... |
| | 21 | 54 | 64 | 53 | —$^{7}$ | — | —$^{8}$ | 6 | 17 | 7 | | | | N .. E .. F ... — .... — .... |
| | 22 | 53 | 62 | 53 | —$^{8}$ | — | —$^{9}$ | — | 10 | — | 1 | — | 4 | NE .. F ... — . R . F .... |
| | 23 | 56 | 60 | 56 | —$^{9}$ | — | —$^{8}$ | | | | 11 | 15 | 30 | NW ... R . C ... — .... |
| | 24 | 56 | 68 | 63 | —$^{7}$ | — | — | | | | 38 | 30 | 26 | NW .. R . — .. F ... — .... |
| | 25 | 62 | 75 | 65 | —$^{8}$ | — | —$^{9}$ | | | | 24 | 1 | 5 | E .. F ... — .... —.... |
| | 26 | 65 | 75 | 63 | —$^{9}$ | — | — | | | | 15 | 2 | 20 | E.. F .... — .... R ... in n ... |

Quantity of Rain from May 27 to June 26, 1 inch and $\frac{34}{100}$.

The estimation of the quantity of Rain, by the Rain Gage, seems liable to some uncertainty, or the quantity falling at places not far distant, may vary exceedingly. In the last month the quantity, where this diary is kept in London, was 2 inches $\frac{38}{100}$, and at Highgate it is stated to have been 5 inches $\frac{50}{100}$.

2d. Variable winds from East to South and West, on the 5th varying in an equal degree; on this day there was a heavy fall of rain.

8th. The thermometer at 80 in the shade at four P. M. Thunder in the evening with rain.

The atmosphere has been generally dry in June, at least in the middle of the month; and the weather was dry and warm, before the hygrometer shewed the decreasing humidity, but which at no period did arise to the same degree as in the beginning of April.

*Princes Street, Cavendish Square*

